# Only Children Were Associated with Anxiety and Depressive Symptoms among College Students in China

**DOI:** 10.3390/ijerph17114035

**Published:** 2020-06-05

**Authors:** Shuo Cheng, Cunxian Jia, Yongjie Wang

**Affiliations:** School of Public Health, Cheeloo College of Medicine, Shandong University, Jinan 250012, China; chengshuo316@163.com (S.C.); jiacunxian@sdu.edu.cn (C.J.)

**Keywords:** college students, only children, anxiety symptom, depressive symptom, China

## Abstract

This study explored the prevalence of anxiety and depressive symptoms among college students and analyzed the associations between only children and anxiety and depressive symptoms in college students in China. A total of 645 college students, from three universities in Jinan, Shandong, China, were investigated by questionnaire. The self-designed general information questionnaire was used to collect the demographic information such as gender, age, only children or not and so on. The Self-rating Anxiety Scale and Self-rating Depression Scale were used to reflect the psychological state of college students. Binary logistic regression analysis was applied to analyze associated factors of anxiety and depressive symptoms. We have found that there were 25.7% college students with anxiety symptom, 22.2% college students with depressive symptom, and 18.3% college students with a comorbidity of anxiety and depressive symptoms. The prevalence of anxiety symptom, depressive symptom, and comorbidity of anxiety and depressive symptoms in only children was higher than those among non-only children. There were no differences between males and females in anxiety symptom, depressive symptom, and comorbidity of anxiety and depressive symptoms among all college students, only child college students and non-only child college students. Only children were associated with anxiety symptom, depressive symptom, comorbidity of anxiety and depressive symptoms after adjusting potential important confounding factors, such as professional category, grade, parental relationship, parenting style, interpersonal relationship, activity participation enthusiasm, sleeping time, and eating habits. The prevalence of anxiety and depressive symptoms among college students was high. We should pay more attention to the mental health of college students, especially that of only child college students.

## 1. Introduction

Anxiety and depression are common mental diseases. People with anxiety are nervous, anxious, or avoid the perception of environmental threats (such as social situations or unfamiliar position) or their own inner selves (such as unusual physical sensations) [1]. Anxiety is highly prevalent in the general population and associated with impairment, disability, and neuropsychological deficits [2,3]. Anxiety is the main cause of disability-adjusted life years worldwide [4,5]. Huang, Y. et al. [6] have found that the prevalence rate of anxiety disorder is 7.6% across the lifetime and 5.0% in the past 12 months in a representative survey of mental diseases in China.

Individuals with depression show a significant and lasting depressed mood, decreased interest or lack of interest in daily activities, insomnia, and an inability to enjoy life [7,8]. The World Health Organization has predicted that depression will rank as the first cause of the burden of disease worldwide by 2030 [9]. In China, the prevalence rate of depression is 6.8% in lifetime and 3.6% in the past 12 months in a representative survey of mental diseases in China [6]. The burden of depression has increased and gradually become a major public health issue in China [10].

College students are the key force that determines the economic growth and success of a country. The time at college is a critical period from adolescence to adulthood [11]. College students are more likely to be susceptible to anxiety, depression, and other mental diseases due to the changes in social roles, decreased social support, and increased pressure [12,13]. In recent years, numerous studies have found that the rates of anxiety and depressive symptoms among college students are significantly higher than in the general population [14,15]. Anxiety and depression have a profound negative impact on the development of college students, not only for academic achievement [16], but also for physical health [17], or even make them prone to negative events such as suicidal ideation or behavior [18], causing irreparable damage to family, country, and society.

China implemented the one-child policy from 1979 which ended in 2016. According to the sixth census in 2010, the number of only children was 164 million and was rising [19,20]. Previous studies have estimated that the number of only children in China would reach 303 million in 2050 [21]. Several researches show that only children are often self-centered, less cooperative, and less likely to get on with their peers [22]. Compared with non-only children, only children are more prone to pessimism or serious psychological problems [23].

In the past several years, many surveys were carried out to explore the mental health of only children among young children and adolescents. However, few studies focused on only children among adults in China [24]. Although some studies have investigated the prevalence of anxiety symptom or depressive symptom separately among college students [25,26], there is little evidence on the prevalence of comorbidity of anxiety and depressive symptoms among college students. So the aims of our study are as follows: (1) to estimate the prevalence rates of anxiety symptom, depressive symptom, comorbidity of anxiety and depressive symptoms among college students and by gender; (2) to compare the different prevalence rates of anxiety symptom, depressive symptom, and comorbidity of anxiety and depressive symptoms between only children and non-only children among college students in total and by gender, respectively; and (3) to explore the associations between only children and anxiety symptom, depressive symptom, and comorbidity of anxiety and depressive symptoms among college students after adjusting some potential confounding factors.

## 2. Materials and Methods

### 2.1. Participants and Data Collection Procedure

A cross-sectional study was conducted in three comprehensive universities in Jinan, Shandong Province, China. We selected one major from three major categories of science and technology, literature and history, and medicine. All classes in each major were numbered, then we selected one class from each grade randomly, from freshman to junior in science and technology and literature and history, from freshman to senior in medicine. All the students in the selected classes were invited to attend this survey in the study. A total of 733 students were invited, of which 654 students (89.2%) agreed to participate. Before the survey, we obtained the informed consent from the subjects, and the unified trained investigators explained some standard requirements for filling in the questionnaire. After completion of the questionnaires, we collected carefully checked questionnaires. In this study, we recovered 645 valid questionnaires. The effective response rate was 88.0%.

### 2.2. Measures

The questionnaire consists of three parts, including a self-designed general information questionnaire, Self-rating Anxiety Scale (SAS), and Self-rating Depression Scale (SDS).

#### 2.2.1. General Social Demographic Characteristics

A self-designed general information questionnaire was used to investigate demographic and social characteristics. The questionnaire included whether he/she was only child (0 = no and 1 = yes), his/her professional category (0 = literature and history, 1 = science and technology, 2 = medicine), grade (0 = sophomore, 1 = freshman, 2 = junior and senior), gender (0 = male and 1 = female), personality (0 = extravert, 1 = moderate, 2 = introvert), physical condition (0 = healthy and 1 = invalid), his/her parental relationship (0 = harmony, 1 = frequent quarrel, 2 = family violence, 3 = parental divorce), his/her parenting style (0 = democratic parenting style and 1 = authoritarian parenting style), his/her activity participation enthusiasm (0 = active, 1 = moderate, 2 = inactive), interpersonal relationship (0 = harmonious and 1 = unharmonious), sleeping time (0 = before 00:00, 1 = 00:00–2:00 am, 2 = after 2:00 am), and eating habits (0 = regular, 1 = irregular).

#### 2.2.2. The Assessment of Anxiety Symptom

Anxiety symptom was measured with the 20-item Self-rating Anxiety Scale (SAS) developed by Zung [27]. The SAS has been widely used among Chinese college students [28,29]. According to the feelings of the subjects in the last week, each item was scored on a four-point scale (1 = none or a little of the time, 2 = some of the time, 3 = good part of the time, 4 = most or all of the time). Five negative items of the SAS utilize reverse scoring. The scores of all items in the scale are added up to a rough score, which is taken as the standard score after × 1.25. The higher the score, the higher the degree of anxiety is. The standard score of SAS ≥ 50 indicates anxiety symptom, which include mild anxiety symptom scored from 50 to 59, moderate anxiety symptom scored from 60 to 69, and severe anxiety symptom scored above 70 [30]. In this study, the Cronbach’s value of SAS was 0.81.

#### 2.2.3. The Assessment of Depressive Symptom

The 20-item Self-rating Depression Scale (SDS) developed by Zung [31] was used to evaluate depressive symptom. The SDS has been widely used among Chinese college students [32,33]. According to the feelings of the subjects in the last week, each item was scored on a four-point scale (1 = none or a little of the time, 2 = some of the time, 3 = good part of the time, 4 = most or all of the time). Ten negative items of SDS utilize reverse scoring. The scores of all items in the scale are added up to a rough score, which is taken as the standard score after × 1.25. The higher the score, the higher the degree of depression is. The standard score of SDS ≥ 53 indicates depressive symptoms, which include mild depressive symptom scored 53 to 62, moderate depressive symptom scored from 63 to 72, and severe depressive symptom scored above 73 [34]. In this study, the Cronbach’s value of SDS was 0.80.

### 2.3. Statistical Analysis

Data were inputted using EpiData 3.1 (The EpiData Association, Odense, Denmark) and analyzed by SPSS 19.0 for Windows (IBM Corp, Armonk, New York, USA). When describing the general demographic characteristics, continuous variables were described by mean (Standard Deviation, SD), and the classification variables were denoted by *n* (%). The chi-square test was used to compare the differences of anxiety and depressive symptom between groups, and the binary logistic regression models were used to analyze the associations between only children and anxiety symptom, depressive symptom, comorbidity of anxiety and depressive symptoms, respectively. *p* < 0.05 was considered to be statistically significant.

## 3. Results

### 3.1. Demographic Characteristics of the Participants

In this study, a total of 645 college students from three universities were investigated. There were 335 (51.9%) only children and 310 (48.1%) non-only children. There were 289 (44.8%) male students and 356 (55.2%) female students. The most frequent of the major categories composition is the professional category of literature and history (41.1%), followed by the professional category of science and technology (39.5%), and medicine (19.4%). In terms of grade composition, there were 200 (31.0%) freshmen, 200 (31.0%) sophomores, and 245 (38.0%) juniors and seniors.

### 3.2. Distribution of Anxiety and Depressive Symptoms among College Students in Total and by Gender

Among the surveyed 645 college students, the standard score of SAS ranged from 25 to 67, with an average score of (43 ± 9). Among the subjects, 166 (25.7%) college students had anxiety symptom, including 134 (20.8%) with mild anxiety symptom and 32 (5.0%) with moderate anxiety symptom. There were 77 male college students with anxiety symptom, accounting for 26.6%, while there were 89 (25.0%) female college students with anxiety symptom.

The standard score of SDS ranged from 25 to 67, with an average score of (45 ± 9). In total, 143 (22.2%) subjects had depressive symptom, among which 129 (20.0%) had mild depressive symptom and 14 (2.2%) had moderate depressive symptom. There were 72 male college students with depressive symptom, accounting for 24.9%, while there were 71 (19.9%) female college students with depressive symptom.

Additionally, there were 118 college students with a comorbidity of anxiety and depressive symptoms, accounting for 18.3%. There were 55 male college students with a comorbidity of anxiety and depressive symptoms, accounting for 19.0%, while there were 63 (17.7%) female college students with comorbidity of anxiety and depressive symptoms.

### 3.3. Comparison of Anxiety and Depressive Symptoms between Only Children and Non-Only Children in Total and by Gender

**Figure 1:**
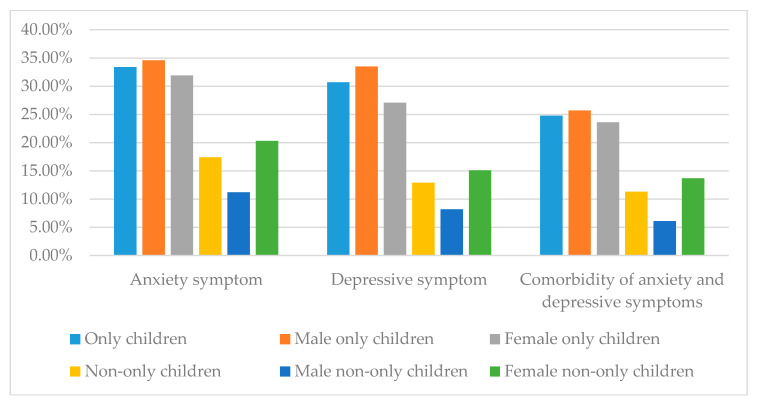
The prevalence rates of anxiety symptom, depressive symptom, comorbidity of anxiety and depressive symptoms among only children, non-only children and by gender.

There were 112 only child college students with anxiety symptom, accounting for 33.4% of the total number of only children. There were 66 male only children with anxiety symptom, accounting for 34.6% of the total number of male only children. There were 46 female only children with anxiety symptom, accounting for 31.9% of the total number of female only children. Among non-only children, 54 of them had anxiety symptom, accounting for 17.4% of the total number of non-only children. Among male non-only children, 11 had anxiety symptom, accounting for 11.2% of the total number of male non-only children. Among female non-only children, 43 had anxiety symptom, accounting for 20.3% of the total number of female non-only children. The results of the chi-square test showed that the difference in anxiety symptom between only children and non-only children was statistically significant. There was no statistical difference between male only children and female only children in anxiety symptom. There was also no statistical difference between male non-only children and female non-only children in anxiety symptom. More details are shown in Figure 1, Table 1 and Table 2.

Among only children, 103 had depressive symptom, accounting for 30.7% of the total number of only children. Among male only children, 64 had depressive symptom, accounting for 33.5% of the total number of male only children. Among female only children, 39 had depressive symptom, accounting for 27.1% of the total number of female only children. There were 40 non-only children with depressive symptom, accounting for 12.9%. Among male non-only children, eight had depressive symptom, accounting for 8.2% of the total number of male non-only children. There were 32 female non-only children with depressive symptom, accounting for 15.1% of the total number of female non-only children. The results of the chi-square test showed that the difference in depressive symptom between only children and non-only children was statistically significant. There was no statistical difference between male only children and female only children in depressive symptom. Additionally, there was also no statistical difference between male non-only children and female non-only children in depressive symptom. More details are shown in Figure 1, Table 1 and Table 2.

There were 83 only child college students with a comorbidity of anxiety and depressive symptoms, accounting for 24.8%. Among male only children, 49 had a comorbidity of anxiety and depressive symptoms, accounting for 25.7% of the total number of male only children. Among female only children, 34 had a comorbidity of anxiety and depressive symptoms, accounting for 23.6% of the total number of female only children. There were 35 non-only children with a comorbidity of anxiety and depressive symptoms, accounting for 11.3%. Among male non-only children, six had a comorbidity of anxiety and depressive symptoms, accounting for 6.1% of the total number of male non-only children. There were 29 female non-only children with a comorbidity of anxiety and depressive symptoms, accounting for 13.7% of the total number of female non-only children. There was a statistically significant difference in the proportion of comorbidity of anxiety and depressive symptoms between only children and non-only children. There was no statistical difference between male only children and female only children in the comorbidity of anxiety and depressive symptoms. There was also no statistical difference between male non-only children and female non-only children in the comorbidity of anxiety and depressive symptoms. More details are shown in Figure 1, Table 1 and Table 2.

### 3.4. Associations between Only Children and Anxiety Symptom, Depressive Symptom, Comorbidity of Anxiety and Depressive Symptoms

The chi-square test indicated that only children were significantly associated with anxiety symptom, depressive symptom, and the comorbidity of anxiety and depressive symptoms in college students (*p_s_* < 0.05), respectively. Other confounding factors such as professional category, grade, parental relationship, parenting style, interpersonal relationship, activity participation enthusiasm, sleeping time, and eating habits were related to anxiety symptom, depressive symptom, comorbidity of anxiety and depressive symptoms (*p_s_* < 0.05). Gender, personality, and physical condition were not statistically significant when compared to the distribution of anxiety symptom, depressive symptom, and the comorbidity of anxiety and depressive and symptoms (*p_s_* > 0.05). Further information is shown in Table 2.

Significant factors in univariate analyses from different comparisons above were included in the binary logistic regression models. In consideration of the important effect of gender on anxiety and depressive symptoms among college students [35,36,37,38], gender was also included in the above models. Multi-factor analyses indicated that only children were associated with anxiety symptom, depressive symptom, comorbidity of anxiety and depressive symptoms (*p* < 0.05) after adjusting confounding factors. More details can be seen in Table 3.

Individuals who were only children (OR = 2.25, *p* < 0.001) were more likely to have anxiety symptom. Freshmen (OR = 1.89, *p* = 0.027), juniors, and seniors (medicine) (OR = 2.95, *p* < 0.001), the professional category of science and technology (OR = 1.68, *p* = 0.041), the professional category of medicine (OR = 2.96, *p* < 0.001), individuals who were inactive in activity participation (OR = 2.52, *p* = 0.001), slept after 2:00 am (OR = 6.09, *p* = 0.014), whose parents had family violence (OR = 8.58, *p* = 0.021), an authoritarian parenting style (OR = 2.53, *p* < 0.001), an unharmonious interpersonal relationship (OR = 2.57, *p* = 0.026), or irregular eating habits (OR = 2.49, *p* < 0.001) were also more likely to have anxiety symptom.

Individuals who were only children (OR = 2.69, *p* < 0.001) were more likely to have depressive symptom. Juniors and seniors(medicine) (OR = 2.63, *p* = 0.001), the professional category of science and technology (OR = 2.78, *p* < 0.001), the professional category of medicine (OR = 2.20, *p* = 0.012), individuals who were inactive in activity participation (OR = 2.47, *p* = 0.002), slept after 2:00 am (OR = 13.02, *p* = 0.003), whose parents had family violence (OR = 10.13, *p* = 0.017), an authoritarian parenting style (OR = 2.58, *p* < 0.001), an unharmonious interpersonal relationship (OR = 2.61, *p* = 0.031), or irregular eating habits (OR = 2.94, *p* < 0.001) were also more likely to have depressive symptom.

Individuals who were only children (OR = 2.37, *p* = 0.001) were more likely to have comorbidity of anxiety and depressive symptoms. Juniors and seniors (medicine) (OR = 2.99, *p* = 0.001), the professional category of medicine (OR = 2.65, *p* = 0.002), individuals who were inactive in activity participation (OR = 2.12, *p* = 0.015), slept after 2:00 am (OR = 8.04, *p* = 0.005), whose parents had family violence (OR = 6.92, *p* = 0.033), an authoritarian parenting style (OR = 2.89, *p* < 0.001), an unharmonious interpersonal relationship (OR = 3.27, *p* = 0.008), irregular eating habits (OR = 3.53, *p* < 0.001) were also more likely to have comorbidity of anxiety and depressive symptoms.

## 4. Discussion

In this study, the major findings are as follows: (1) there were 25.7% college students with anxiety symptom, 22.2% college students with depressive symptom, and 18.3% college students with comorbidity of anxiety and depressive symptoms. There were no statistical differences in anxiety symptom, depressive symptom, and comorbidity of anxiety and depressive symptoms between males and females among college students; (2) the prevalence rates of anxiety symptom, depressive symptom and the comorbidity of anxiety and depressive symptoms among only children were higher than those among non-only children. There were no statistical differences between male only children and female only children in anxiety symptom, depressive symptom, and comorbidity of anxiety and depressive symptoms. Additionally, there were no statistical differences between male non-only children and female non-only children in anxiety symptom, depressive symptom, and comorbidity of anxiety and depressive symptoms; (3) only children were associated with anxiety symptom, depressive symptom, and comorbidity of anxiety and depressive symptoms after adjusting some potential confounders.

College time is a crucial period of life for students, a college students’ mindset is not only related to their academic achievement, but also the progress and development of the society in the future. After entering college, they are easily affected by various factors such as major choice, interpersonal communication, family atmosphere, living habits, and so on, resulting in various psychological problems such as anxiety and depression [39,40,41]. Of all college students, 25.7% had anxiety symptom in this study, which is higher than recent research in China (15.4%–17.4%) [42]. Lei, X.Y. et al. [43] reported a higher rate (23.8%) of depressive symptom among college students compared with our findings (22.2%), which is higher than the findings from Tang, F. et al. (19.6%) [44] and Yang, X. et al. (8.7%) [45].

We found only children were associated with anxiety symptom, depressive symptom, comorbidity of anxiety and depressive symptoms, which is consistent with the results of Li Dan et al. [46]. There are some reasons regarding personality to explain these associations. Firstly, only children are more stubborn than non-only children, and have a relatively poor ability to adapt to the environment [47]. Secondly, only children are more likely to avoid difficulties and problems than non-only children. In addition, only children have low self-esteem, sense of security, are less happy, and show high levels of depression [48].

There was no statistically significant difference in the proportion of anxiety symptom and depressive symptom in college students between males and females, which was consistent with the study from Wu, S. et al. [49]. Furthermore, there were no statistical differences between males and females in anxiety symptom, depressive symptom, or comorbidity of anxiety and depressive symptoms in only children and non-only child group, respectively.

In this study, grade, professional category, parental relationship, parenting style, interpersonal relationship, activity participation enthusiasm, sleeping time, and eating habits were also found to be associated with anxiety symptom, depressive symptom, and the comorbidity of anxiety and depressive symptoms. Compared with sophomores, freshmen were more likely to have anxiety symptom. This agrees with other results [50,51]. Juniors and seniors (medical science) were prone to anxiety symptom, depressive symptom, and the comorbidity of anxiety and depressive symptoms. The result is consistent with Wu, D. et al.’s study [52] and this may be related to the increased stress in the later period of the university. Studies have confirmed that academic stress was positively associated with negative emotions [53]. Compared with literature and history majors, medical majors were more prone to anxiety symptom, depressive symptom, and the comorbidity of anxiety and depressive symptoms, in line with other findings [54,55,56,57,58]. The study pressure of medical college students is greater than that of other professions because their course content is more difficult to understand [59]. Furthermore, medical education is especially rigorous and has numerous higher requirements than other majors, as a profession related to the safety of human life [60]. This study revealed that college students whose parents often choose authoritarian parenting style were prone to anxiety symptom, depressive symptom, and comorbidity of anxiety and depressive symptoms, compared with those whose parents often choose democratic parenting style. College students whose parental relationship displays family violence were prone to anxiety symptom, depressive symptom, and the comorbidity of anxiety and depressive symptoms, compared with those whose parental relationship is harmonious. This can be explained by other results. Parents raised their children in positive ways such as warmth, affirmation, and support, and the more they paid attention to their children’s emotions, thoughts, and behaviors, make timely and appropriate responses, the better their psychological health was [61]. Children raised in an atmosphere of parental domestic violence were associated with increased negative effects, including pain, fear, anger and worry, and heightened emotions sensitivity [62]. The enthusiasm of participating in activities and interpersonal relationship were associated with the anxiety and depressive symptoms of college students. This result is in line with the interpersonal relationship theory of depression [63]. Good interpersonal relationships enable college students to obtain more spiritual support and help, thus reducing the occurrence of anxiety and depression [64]. This study showed that eating habits and sleeping time were associated with anxiety symptom, depressive symptom, and the comorbidity of anxiety and depressive symptoms. Irregular eating habits cause many body diseases, having a negative impact on health [65]. Healthy eating habits may increase their likelihood of getting adequate sleep [66]. A meta-analysis of the relationship between Chinese college students’ sleep quality and depression found that college students stayed up late would result in a worse sleep quality, having a higher risk of producing the depressed mood [67].

There are several limitations in our study. First, the sample of this study was 645 college students from three universities in Jinan, the extrapolation of the results may be limited, and a larger investigation should be carried out in the future. Second, mental health was assessed by self-reported in this study. Although these instruments have been validated and have acceptable sensitivity and specificity in college students, it could lead to reporting bias due to forgetting information or unwillingness to disclose information. Third, it was a cross-sectional survey, and a cause-and-effect relationship was not concluded. Future research with the longitudinal design based on a large and representative sample is needed to confirm the factors related to the anxiety and depressive symptoms of college students.

## 5. Conclusions

The prevalence rates of anxiety symptom, depressive symptom, and comorbidity of anxiety and depressive symptoms among college students were high, especially in only children. There were no differences between males and females in anxiety symptom, depressive symptom, and comorbidity of anxiety and depressive symptoms among all college students, only child college students and non-only child college students. Only children were associated with anxiety symptom, depressive symptom, and comorbidity of anxiety and depressive symptoms after adjusting potential and important confounding factors. We should pay more attention to the mental health of college students especially only child college students. Intervention measures should be considered for mental health in college students especially only children.

## Figures and Tables

**Table 1:** Comparison of anxiety and depressive symptoms between males and females by only children or not.

Variables	Total		*χ^2^*	*p*	Only Children		*χ^2^*	*p*	Non-Only Children		*χ^2^*	*p*
	Male *n* (%)	Female *n* (%)			Male *n* (%)	Female *n* (%)			Male *n* (%)	Female *n* (%)		
Anxiety symptom	77 (26.6)	89 (25.0)	0.23	0.635	66 (34.6)	46 (31.9)	0.25	0.616	11 (11.2)	43 (20.3)	3.82	0.051
Depressive symptom	72 (24.9)	71 (19.9)	2.28	0.131	64 (33.5)	39 (27.1)	1.59	0.207	8 (8.2)	32 (15.1)	2.87	0.091
Comorbidity of anxiety and depressive symptoms	55 (19.0)	63 (17.7)	0.19	0.663	49 (25.7)	34 (23.6)	0.18	0.668	6 (6.1)	29 (13.7)	3.82	0.051

**Table 2:** Univariate analysis of associated factors of anxiety and depressive symptoms among college students.

Variables	Anxiety Symptom			Depressive Symptom			Comorbidity of Anxiety and Depressive Symptoms		
	*n* (%)	*χ^2^*	*p*	*n* (%)	*χ^2^*	*p*	*n* (%)	*χ^2^*	*p*
Only children or not		21.60	<0.001		29.71	<0.001		19.59	<0.001
Yes	112 (33.4)			103 (30.7)			83 (24.8)		
No	54 (17.4)			40 (12.9)			35 (11.3)		
Professional category		15.00	0.001		18.60	<0.001		9.89	0.007
Science and technology	72 (28.2)			75 (29.4)			53 (20.8)		
Literature and history	49 (18.5)			37 (14.0)			34 (12.8)		
Medicine	45 (36.0)			31 (24.8)			31 (24.8)		
Grade		23.36	<0.001		17.23	<0.001		17.38	<0.001
Freshman	50 (25.0)			41 (20.5)			34 (17.0)		
Sophomore	30 (15.0)			28 (14.0)			21 (10.5)		
Junior and senior	86 (35.1)			74 (30.2)			63 (25.7)		
Gender		0.23	0.635		2.28	0.131		0.19	0.663
Male	77 (26.6)			72 (24.9)			55 (19.0)		
Female	89 (25.0)			71 (19.9)			63 (17.7)		
Personality		1.75	0.418		4.32	0.115		4.25	0.120
Extravert	56 (22.9)			44 (18.0)			35 (14.3)		
Moderate	91 (27.3)			84 (25.2)			69 (20.7)		
Introvert	19 (28.4)			15 (22.4)			14 (20.9)		
Physical condition		0.13	0.720		0.39	0.534		0.91	0.340
Healthy	163 (25.6)			140 (22.0)			115 (18.1)		
Invalid	3 (37.5)			3 (37.5)			3 (37.5)		
Parental relationship		10.83	0.009		12.33	0.004		10.33	0.011
Harmony	91 (22.4)			81 (20.0)			64 (15.8)		
Frequent quarrel	67 (29.9)			53 (23.7)			47 (21.0)		
Family violence	4 (66.7)			4 (66.7)			3 (50.0)		
Parental divorce	4 (44.4)			5 (55.6)			4 (44.4)		
Parenting style		25.38	<0.001		21.97	<0.001		24.59	<0.001
Authoritarian parenting style	69 (40.1)			60 (34.9)			65 (30.8)		
Democratic parenting style	97 (20.5)			83 (17.5)			53 (13.7)		
Activity participation enthusiasm		39.75	<0.001		35.22	<0.001		34.98	<0.001
Active	50 (20.0)			41 (16.4)			36 (14.4)		
Moderate	60 (21.4)			53 (18.9)			39 (13.9)		
Inactive	56 (49.1)			49 (43.0)			43 (37.7)		
Interpersonal relationship		7.87	0.005		9.09	0.003		11.23	0.001
Harmonious	151 (24.6)			129 (21.0)			105 (17.1)		
Unharmonious	15 (46.9)			14 (43.8)			13 (40.6)		
Sleeping time		24.61	<0.001		35.34	<0.001		35.35	<0.001
Before 00:00	95 (22.2)			82 (19.2)			69 (16.2)		
00:00–2:00 am	60 (29.4)			49 (24.0)			38 (18.6)		
After 2:00 am	11 (78.6)			12 (85.7)			11 (78.6)		
Eating habits		28.64	<0.001		32.78	<0.001		37.11	<0.001
Regular	111 (21.3)			92 (17.6)			72 (13.8)		
Irregular	55 (44.7)			51 (41.5)			46 (37.4)		

**Table 3:** Logistic regression analysis of association between only children and anxiety symptom, depressive symptom, comorbidity of anxiety and depressive symptoms.

Variables	Anxiety Symptom			Depressive Symptom			Comorbidity of Anxiety and Depressive Symptoms		
	OR	95% CI	*p*	OR	95% CI	*p*	OR	95% CI	*p*
Only children or not									
Yes	2.25	1.47–3.46	<0.001	2.69	1.69–4.30	<0.001	2.37	1.44–3.90	0.001
No	1.00	-	-	1.00	-	-	1.00	-	-
Professional category									
Science and technology	1.68	1.02–2.77	0.041	2.78	1.62–4.77	<0.001	1.75	0.99–3.11	0.055
Literature and history	1.00	-	-	1.00	-	-	1.00	-	-
Medicine	2.96	1.71–5.12	<0.001	2.20	1.19–4.08	0.012	2.65	1.41–4.97	0.002
Grade									
Freshman	1.89	1.08–3.33	0.027	1.48	0.81–2.71	0.204	1.68	0.86–3.26	0.127
Sophomore	1.00	-	-	1.00	-	-	1.00	-	-
Junior and senior	2.95	1.74–5.02	<0.001	2.63	1.50–4.61	0.001	2.99	1.61–5.55	0.001
Gender									
Male	1.00	-	-	1.00	-	-	1.00	-	-
Female	0.99	0.62–1.58	0.965	1.04	0.63–1.72	0.873	0.98	0.57–1.68	0.947
Parental relationship									
Harmony	1.00	-	-	1.00	-	-	1.00	-	-
Frequent quarrel	1.18	0.76–1.82	0.455	1.00	0.63–1.59	0.993	1.09	0.66–1.79	0.736
Family violence	8.58	1.38–53.25	0.021	10.13	1.52–67.45	0.017	6.92	1.17–40.84	0.033
Parental divorce	1.15	0.24–5.53	0.862	3.53	0.72–17.16	0.119	2.26	0.46–11.16	0.319
Parenting style									
Authoritarian parenting style	2.53	1.63–3.93	<0.001	2.58	1.62–4.12	<0.001	2.89	1.77–4.72	<0.001
Democratic parenting style	1.00	-	-	1.00	-	-	1.00	-	-
Activity participation enthusiasm									
Active	1.00	-	-	1.00	-	-	1.00	-	-
Moderate	0.93	0.58–1.49	0.765	1.04	0.63–1.74	0.875	0.76	0.44–1.32	0.333
Inactive	2.52	1.46–4.35	0.001	2.47	1.38–4.42	0.002	2.12	1.16–3.87	0.015
Interpersonal relationship									
Harmonious	1.00	-	-	1.00	-	-	1.00	-	-
Unharmonious	2.57	1.12–5.92	0.026	2.61	1.09–6.24	0.031	3.27	1.35–7.88	0.008
Sleeping time									
Before 00:00	1.00	-	-	1.00	-	-	1.00	-	-
00:00–2:00 am	1.15	0.74–1.79	0.526	1.01	0.63–1.61	0.970	0.83	0.50–1.39	0.480
After 2:00 am	6.09	1.43–25.85	0.014	13.02	2.46–68.92	0.003	8.04	1.86–37.72	0.005
Eating habits									
Regular	1.00	-	-	1.00	-	-	1.00	-	-
Irregular	2.49	1.51–4.09	<0.001	2.94	1.75–4.96	<0.001	3.53	2.05–6.10	<0.001
